# Biases in the Experimental Annotations of Protein Function and Their Effect on Our Understanding of Protein Function Space

**DOI:** 10.1371/journal.pcbi.1003063

**Published:** 2013-05-30

**Authors:** Alexandra M. Schnoes, David C. Ream, Alexander W. Thorman, Patricia C. Babbitt, Iddo Friedberg

**Affiliations:** 1 Department of Bioengineering and Therapeutic Sciences, University of California, San Francisco, San Francisco, California, United States of America; 2 Department of Microbiology, Miami University, Oxford, Ohio, United States of America; 3 Department of Computer Science and Software Engineering, Miami University, Oxford, Ohio, United States of America; University College London, United Kingdom

## Abstract

The ongoing functional annotation of proteins relies upon the work of curators to capture experimental findings from scientific literature and apply them to protein sequence and structure data. However, with the increasing use of high-throughput experimental assays, a small number of experimental studies dominate the functional protein annotations collected in databases. Here, we investigate just how prevalent is the “few articles - many proteins” phenomenon. We examine the experimentally validated annotation of proteins provided by several groups in the GO Consortium, and show that the distribution of proteins per published study is exponential, with 0.14% of articles providing the source of annotations for 25% of the proteins in the UniProt-GOA compilation. Since each of the dominant articles describes the use of an assay that can find only one function or a small group of functions, this leads to substantial biases in what we know about the function of many proteins. Mass-spectrometry, microscopy and RNAi experiments dominate high throughput experiments. Consequently, the functional information derived from these experiments is mostly of the subcellular location of proteins, and of the participation of proteins in embryonic developmental pathways. For some organisms, the information provided by different studies overlap by a large amount. We also show that the information provided by high throughput experiments is less specific than those provided by low throughput experiments. Given the experimental techniques available, certain biases in protein function annotation due to high-throughput experiments are unavoidable. Knowing that these biases exist and understanding their characteristics and extent is important for database curators, developers of function annotation programs, and anyone who uses protein function annotation data to plan experiments.

## Author Summary

Experiments and observations are the vehicles used by science to understand the world around us. In the field of molecular biology, we are increasingly relying on high-throughput, genome-wide experiments to provide answers about the function of biological macromolecules. However, any experimental assay is essentially limited in the type of information it can discover. Here, we show that our increasing reliance on high-throughput experiments biases our understanding of protein function. While the primary source of information is experiments, the functions of many proteins are computationally annotated by sequence-based similarity, either directly or indirectly, to proteins whose function is experimentally determined. Therefore, any biases in experimental annotations can get amplified and entrenched in the majority of protein databases. We show here that high-throughput studies are biased towards certain aspects of protein function, and that they provide less information than low-throughput studies. While there is no clear solution to the phenomenon of bias from high-throughput experiments, recognizing its existence and its impact can help take steps to mitigate its effect.

## Introduction

Functional annotation of proteins is an open problem and a primary challenge in molecular biology today [1]–[4]. The ongoing improvements in sequencing technology have shifted the emphasis from realizing the $1,000 genome to realizing the 1-hour genome [5]. The ability to rapidly and cheaply sequence genomes is creating a flood of sequence data, but to make these data useful, extensive analysis is needed. A large portion of this analysis involves assigning biological function to newly determined gene sequences, a process that is both complex and costly [6]. To aid current annotation procedures and improve computational function prediction algorithms, high-quality, experimentally derived data are necessary. Currently, one of the few repositories of such data is the UniProt-GOA database [7], which is a compilation of data contributed by several member groups of the GO consortium. UniProt-GOA contains functional information derived from literature, and by computational means. The information derived from literature is extracted by human curators who capture functional data from publications, assign the data to their appropriate place in the Gene Ontology hierarchy [8], and label them with appropriate functional evidence codes. UniProt-GOA is compiled from annotations made by several member groups of the GO consortium, and as such presents the current state of our view of protein function space. It is therefore important to understand any trends and biases that are encapsulated in UniProt-GOA, as those impact well-used sister databases and consequently a large number of users worldwide.

One concern surrounding the capture of functional data from articles is the propensity for high-throughput experimental work to become a large fraction of the data in the GO Consortium database, thus having a small number of experiments dominate the protein function landscape. In this work we analyzed the relative contribution of peer-reviewed articles describing all the experimentally derived annotations in UniProt-GOA. We found some striking trends, stemming from the fact that a small fraction of articles describing high-throughput experiments disproportionately contribute to the pool of experimental annotations of model organisms. Consequently we show that: 1) annotations coming from high-throughput experiments are overall less informative than those provided by low-throughput experiments; 2) annotations from high-throughput experiments are biased towards a limited number of functions, and 3) many high-throughput experiments overlap in the proteins they annotate, and in the annotations assigned. Taken together, our findings offer a picture of how the protein function annotation landscape is generated from scientific literature. Furthermore, due to the biases inherent in the current system of sequence annotations, this study serves as a caution to the producers and consumers of biological data from high-throughput experiments.

## Results

### Articles and Proteins

**Figure 1:**
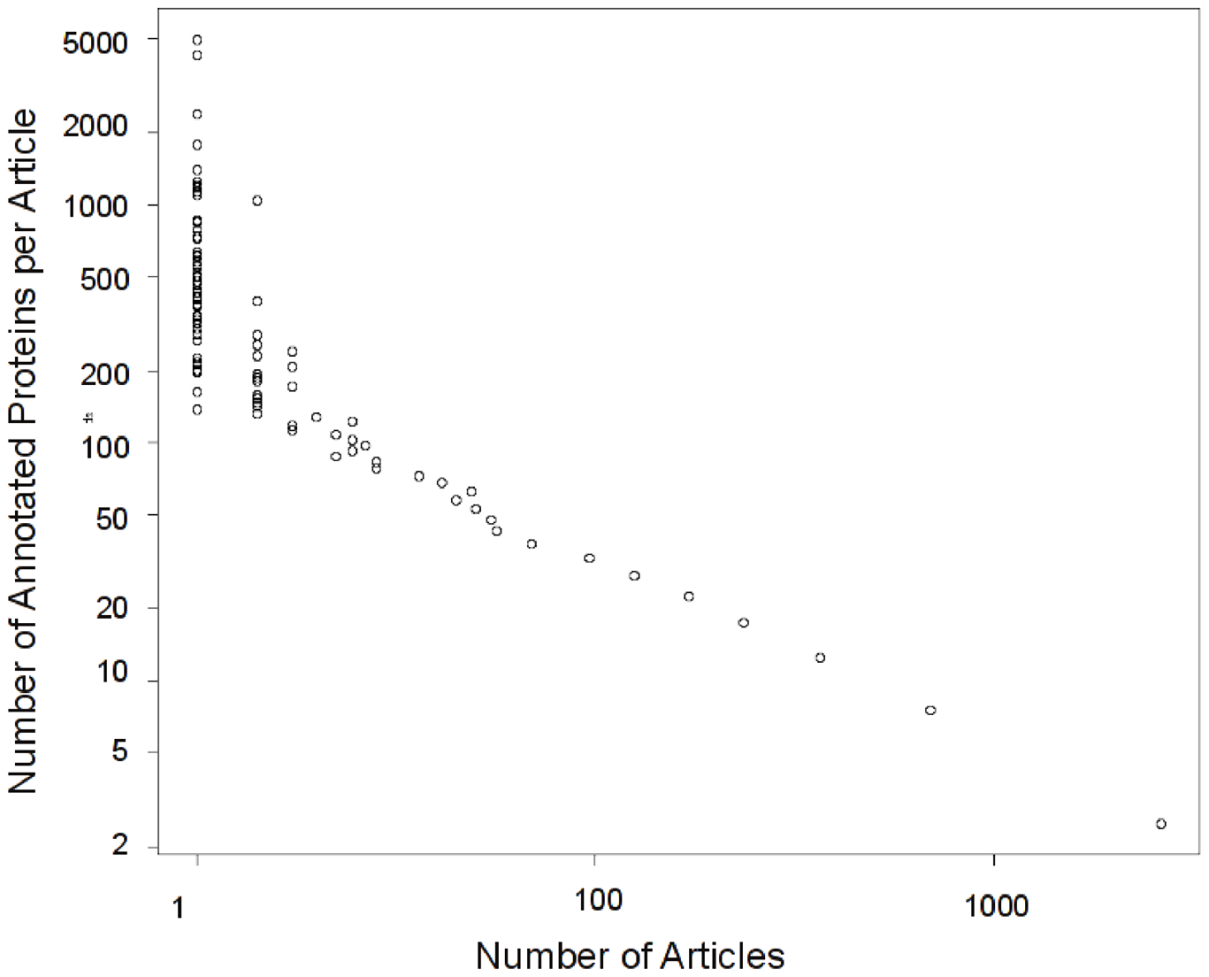
Distribution of the number of proteins annotated per article. X-axis: number of annotating articles. Y-axis: number of annotated proteins. The distribution was found to be logarithmic with a significant (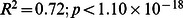) linear fit to the log-log plot. The data came from 76137 articles annotating 256033 proteins with GO experimental evidence codes, in Uniprot-GOA 12/2011.

The increase in the number of high-throughput experiments used to determine protein functions may introduce biases into experimental protein annotations, due to the inherent capabilities and limitations of high-throughput assays. To test the hypothesis that such biases exist, and to study their extent if they do, we compiled the details of all experimentally annotated proteins in UniProt-GOA. This included all proteins whose GO annotations have the GO experimental evidence codes EXP, IDA, IPI, IMP, IGI, IEP (See Methods for an explanation of GO evidence codes). We first examined the distribution of articles that are the source of experimentally validated annotations by the number of proteins they annotate. As can be seen in Figure 1, the distribution of the number of proteins annotated per article follows a power-law distribution. 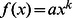. Using linear regression over the log values of the axes we obtained a fit with 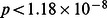 and 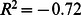. We therefore conclude that there is indeed a substantial bias in experimental annotations, in which there are few articles that annotate a large number of proteins.

To better understand the consequences of such a distribution, we divided the annotating articles into four cohorts, based on the number of proteins each article annotates. *Single-throughput* articles are those articles that annotate only one protein; *low throughput* articles annotate 2–9 proteins; *moderate throughput* articles annotate 10–99 proteins and *high throughput* articles annotate over 99 proteins. The results are shown in Table 1. The most striking finding is that high throughput articles are responsible for 25% of the annotations that the GO Consortium creates, even though they are found only in 0.14% of the articles. 96% of the articles are single-throughput and low-throughput, however those annotate only 53% of the proteins. So while moderate-throughput and high-throughput studies account for almost 47% of the annotations in Uniprot-GOA, they constitute only 3.66% of the studies published.

**Table 1:** Annotation Cohorts.

Articles annotating the following number of proteins	1	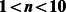	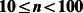	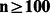	SUM
**Number of proteins annotated**	20699	46383	26485	31411	124978
**Number of annotating articles**	41156	32201	2672	108	76137
**Percent of proteins annotated**	16.56	37.11	21.19	25.13	100
**Percent of annotating articles**	54.09	42.32	3.51	0.14	100

Number of proteins and annotating articles assigned to each article annotation cohort. Columns: 1: articles annotating a single protein (singletons); 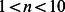 articles annotating more than 1 and less than 10 proteins (low throughput); 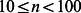: medium throughput; 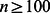: articles annotating 100 proteins and more (high throughput). As can be seen, high-throughput articles comprise 0.14% of the total articles used for experimental annotations, but annotate 25.13% of the proteins in UniProt-GOA.

To understand how the log-odds distribution affects our understanding of protein function, we examined different aspects of the annotations in the four article cohorts. Also, we examined in greater detail the top-50 high-throughput annotating articles. “Top-50 high throughput annotating articles” are the articles describing experimental annotations that are top ranked by the number of proteins annotated per article. An initial characterization of these articles is shown in Table S1 in Text S1. As can be seen, most of the articles are specific to a single species (typically a model organism) and to a single assaying pipeline that is used to assign function to the proteins in that organism. With one exception, only one ontology of the three GO ontologies was used for annotation in any single experiment. The three ontologies are Molecular Function (MF), Biological Process (BP) and Cellular Component (CC). These are separate ontologies within GO, describing different aspects of function as detailed in [8]. As we show later, for some species this means that a single functional aspect (MF, BP or CC) of a species can be dominated by a single study.

### The Impact of High Throughput Studies on the Annotation of Model Organisms

**Figure 2:**
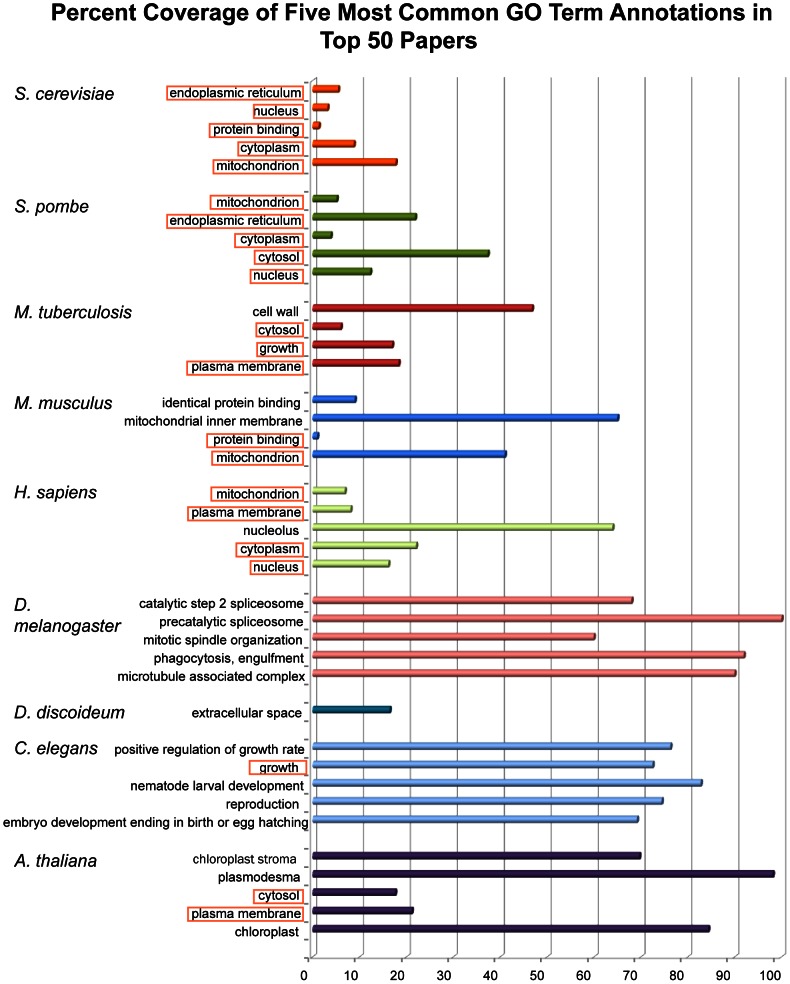
Relative contribution of top-50 articles to the annotation of major model organisms. The length of each bar represents the percentage of proteins annotated by the top-50 articles in a given organism by a given GO term. GO terms that are present in more than one species are highlighted.

We examined the relative contribution of the top-50 articles to the entire corpus of experimentally annotated proteins in each species. Unsurprisingly, all the species found in the top-50 articles were either common model organisms or human. For each species, we examined the five most frequent terms in the top-50 articles. We then examined the contribution of this term by the top-50 articles to the general annotations of that species. The *contribution* is the number of annotations by any given GO term in the top 50 articles divided by the number of annotations by that GO term in all of UniProt-GOA. For example, as seen in Figure 2 in *D. melanogaster*, 88% of the annotations using the term “precatalytic splicosome” in articles experimentally annotating this species that are contributed by the top-50 articles.

For most organisms annotated by the top-50 articles, the annotations were within the Cellular Component or Biological Process ontologies. Notable exceptions are *D. melanogaster* and *C. elegans* where the dominant terms were from the Biological Process ontology, and in mouse, where “protein binding” and “identical protein binding” are from the Molecular Function Ontology. *D. melanogaster*'s annotation for the top terms is dominated (over 50% contribution) by the top-50 articles.

The term frequency bias described here can be viewed more broadly within the ontology bias. The proteins annotated by the cohorts of single-protein articles, low-throughput articles, and moderate-throughput articles have similar ratios of the fraction of proteins annotated. Twenty-two to twenty-six percent of assigned terms are in the Molecular Function Ontology, and 51–57% are in the Biological Process Ontology and the remaining 17–25% are in the Cellular Component ontology. These ratios change dramatically with high-throughput articles (over 99 terms per article). In the high-throughput articles, only 5% of assigned terms are in the Molecular Function Ontology, 38% in the Biological Process Ontology and 57% in the Cellular Compartment Ontology, ostensibly due to a lack of high-throughput assays that can be used for generating annotations using the Molecular Function Ontology.

### Repetition and Consistency in Top-50 Annotations

How many of the top-50 articles actually annotate the same set of proteins? Answering this question will tell us how repetitive experiments are in identifying the same set of proteins to annotate. However, even when annotating the same set of proteins and within the same ontology, different experiments may provide different results, lacking consistency. Therefore, the annotation consistency was also checked. Repetition is given as 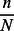 with 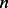 being the number of proteins annotated by two or more articles, and 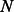 being the total number of proteins.

**Figure 3:**
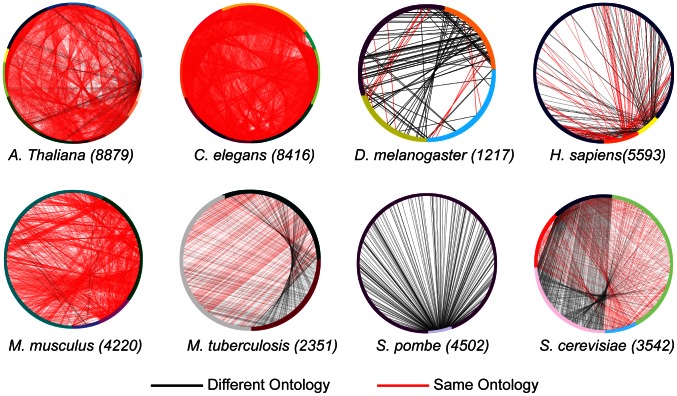
Redundancy in proteins described by the top-50 articles. A circle represents the sum total of articles annotating each organism. Each colored arch is composed of all the proteins in a single article. A line is drawn between any two points on the circle if the proteins they represent have 100% sequence identity. A black line is drawn if they are annotated with a different ontology (for example, in one article the protein is annotated with the MFO, and in another article with BPO); a red line if they are annotated in the same ontology. Example: *S. pombe* is described by two articles, one with few protein (light arch on bottom) and one with many (dark arch encompassing most of circle). Many of the same proteins are annotated by both articles. See Table 2 for numbers.

The results of the repetition analysis are shown in Figure 3 and in Table 2. As can be seen, the highest repetition (65%) is in the 12 articles annotating *C. elegans*. Of course, a higher number of articles is expected to increase repetitive annotations simply due to increased sampling of the genome. However, the goal of this analysis is to present the degree of repetition, rather than to try to rank and normalize it. As an additional repetition metric, Table 2 also lists the mean number of sequences per cluster. When normalized by number of annotating articles, the highest repetition is found in Mouse (15.33% in three articles) closely followed by *M. tuberculosis* (14% in two articles). Taken together, these results show that there is repetition in choosing the proteins that are to be annotated in most model organisms using high-throughput assays, although the rate of this repetition varies widely.

**Table 2:** Sequence Redundancy in Top-50 Annotating Articles.

Species	num. articles	num. prot	Clusters at 100%	% redundancy	Mean genes/cluster
*C. elegans*	12	8416	3338	60	3.74
*A. thaliana*	16	8879	4694	47	3.92
*M. musculus*	3	4220	2273	46	2.75
*M. tuberculosis*	2	2351	1702	28	2.22
*S. cerevisiae*	5	3542	2550	28	2.33
*H. sapiens*	4	5593	4509	19	2.36
*D. melanogaster*	3	1217	1003	18	2.17
*S. pombe*	2	4502	4281	5	2.00

**Species**: annotated species; **num. articles** number of annotating articles; **num. prot**: number of proteins annotated by top-50 articles for that species; **Clusters at 100%**: number of clusters of 100% identical proteins; **% redundancy**: the product of column 4 by column 3: this is the percentage of proteins annotated more than once for a given species in the top 50 articles; **Mean genes/cluster**: the mean number of genes per cluster, for clusters having more than a single gene.

Consistency analysis took place as described in Methods. The consistency measure is normalized on a 0–1 scale, with 1 being most consistent, meaning that all annotations from all sources are identical. Table 3 shows the results of this analysis. In *A. thaliana*, 1941 proteins are annotated by 15 articles and 18 terms in the Cellular Component ontology. The mean maximum-consistency is 0.251. The highest mean consistency is for the annotation of 807 mouse proteins annotated in Cellular Component ontology with an annotation consistency 0.832. However, that is not surprising given that there are only three annotating articles, and two annotating terms. We omitted the ontology and organism combinations that were annotated by less than three articles or two GO terms, or both.

**Table 3:** Annotation Consistency in Top 50 articles.

Species	Ont.	num prot	mean 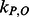	stdv	stderr	num articles	num terms
*A. thaliana*	CCO	1941	0.251	0.328	0.007	15	18
*C. elegans*	BPO	1847	0.388	0.239	0.006	12	41
*D. melanogaster*	BPO	76	0.086	0.22	0.025	3	8
*D. melanogaster*	CCO	81	0.068	0.234	0.026	3	5
*H. sapiens*	CCO	167	0.285	0.365	0.028	2	20
*M. musculus*	CCO	807	0.832	0.291	0.01	3	2
*S. cerevisiae*	CCO	744	0.759	0.379	0.014	4	15
*B. tuberculosis*	CCO	532	0.309	0.41	0.018	2	3

**Species**: annotated species; **Ontology**: annotating GO ontology; **num prot**: number of annotated proteins in that species & ontology that are annotated by more than one paper. **mean, stdv, stderr**: mean number of consistent annotations for a protein in that species and ontology, standard deviation from the mean and standard error. **num articles**: number of annotating articles **num terms** number of annotating terms. Annotations by less than two articles or two terms (or both) for the same protein/ontology combination have been omitted.

### Quantifying Annotation Information

A common assumption holds that while high-throughput experiments annotate more protein functions than low-throughput experiments, the former also tend to be more shallow in the predictions they provide. The information provided, for example, by a large-scale protein binding assay will only tell us if two proteins are binding, but will not reveal whether that binding is specific, will not provide an exact 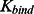, will not say under what conditions binding takes place, or whether there is any enzymatic reaction or signal-transduction involved. Having on hand data from experiments with different “throughputness” levels, we set out to investigate whether there is indeed a difference in the information provided by high-throughput experiments vs. low-throughput ones. We examined the information provided by GO terms in each paper cohort using two methods: edge-count, and information-content. See Methods for details.

**Figure 4:**
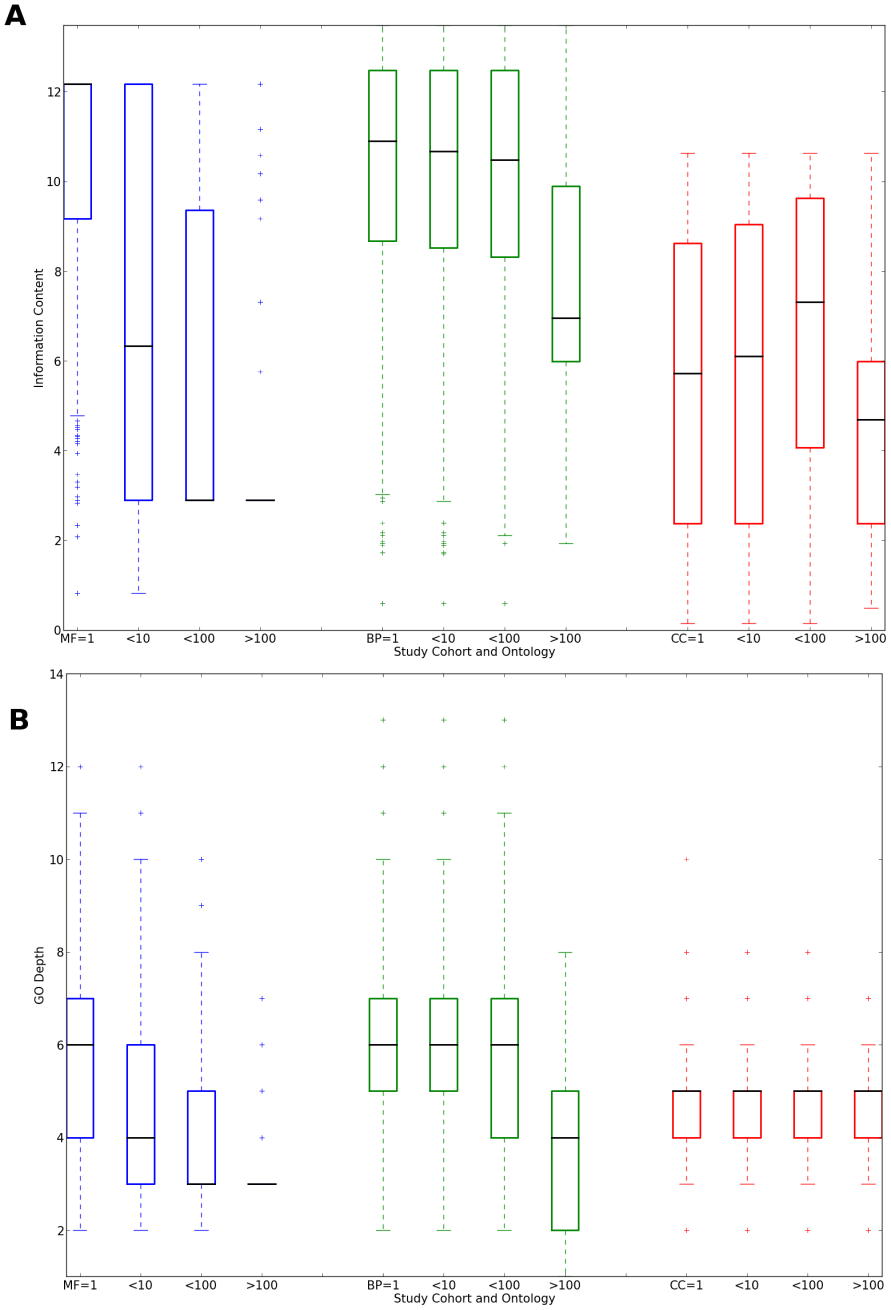
Information provided by articles depending on the number of proteins the articles annotate. Articles are grouped into cohorts: 1: one protein annotated by article; 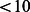: more than 1, up to 10 annotated; 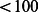: more than 10, less than 100 annotated; 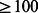: 100 or more proteins annotated per article. Blue bars: Molecular Function ontology; Green bars: Biological Process ontology; Red bars: Cellular Component ontology. Information is gauged by **A**: Information Content and **B**: GO depth. See text for details.

The results of both analyses are shown in Figure 4. In general, the results from the edge count analysis and the information-content based analysis are in agreement when compared across annotation cohorts. For the Molecular Function ontology, the distribution of edge counts and log-frequency scores decreases as the number of annotated proteins per-article increases. For the Biological Process ontology, the decrease is significant. However the contributors to the decrease are the high-throughput articles while there is little change in the first three article cohorts. Finally, there is no significant trend of GO-depth decrease in the Cellular Component Ontology. However, using the information-content metric, there is also a significant decrease in information-content in the high-throughput article cohort.

### Exclusive High Throughput Annotations

Of interest is the fraction of proteins that are exclusively annotated by high-throughput experiments. The question here is: from the experimentally annotated proteins in an organism, how much do we know of their function *only* using high-throughput experiments? We have seen that high-throughput experiments annotate a large number of proteins, but still some 80% of experimentally determined proteins are annotated via medium-, low- and single-throughput experiments. Given the lower information-content of high-throughput experiments, it is important to know which organisms have a substantial fraction of the proteins experimentally annotated by high throughput studies only. To do so, we analyzed all species with more than 200 genes in the NCBI taxa database for the fraction of the genes that are exclusively annotated by high throughput studies. The results are shown in Table 4.

**Table 4:** Fraction of Proteins Exclusively Annotated by High Throughput Studies.

Taxon ID	Taxon	XHT	Total Proteins	%XHT
284812	*S. pombe*	2781	4507	61.704
1773	*B. tuberculosis*	1224	2317	52.8269
6239	*C. elegans*	2493	5302	47.02
9606	*H. sapiens*	4016	11521	34.8581
44689	*D. discoideum*	425	1256	33.8376
3702	*A. thaliana*	3199	10153	31.5079
237561	*C. albicans* SC5314	327	1243	26.3073
10090	*M. musculus* LK3 transgenic	2567	22068	11.6322
7227	*D. melanogaster*	735	7501	9.7987
559292	*S. cerevisiae*	439	5086	8.6315
83333	*E. coli* K-12	83	1606	5.1681
7955	*B. rerio*	117	4633	2.5254
10116	*R. norvegicus*	11	4634	0.2374

**Taxon ID**: NCBI Taxon ID number; **Species**: annotated species; **XHT**: number of proteins exclusively annotated by high-throughput experimental studies (100 or more proteins annotated per study); **Total proteins**: Total number of experimentally annotated proteins in that species. **%XHT**: percentage of proteins in that species that are annotated exclusively by HT studies.

As can be seen, although the fraction of high-throughput annotated proteins is large, not many species are affected with a large fraction of proteins that are exclusively annotated by high-throughput studies. However, the few species that are affected are important study and model species. It is important to note that some redundancy due to isoforms, mutants and duplications may exist.

### Frequently Used High Throughput Experiments

The twenty GO evidence codes, discussed above, encapsulate the means by which the function was inferred, but they do not capture all the necessary information. For example, “Inferred by Direct Assay” (IDA) informs that some experimental assay was used, but does not say which type of assay. This information is often needed, since knowing which experiments were performed can help the researcher establish the reliability and scope of the produced data. RNA, used in an RNAi experiment does not traverse the blood-brain-barrier, meaning that no data from the central nervous system can be drawn from an RNAi experiment. The Evidence Code Ontology, or ECO, seeks to improve upon the GO-attached evidence codes. ECO provides more elaborate terms than “Inferred by Direct Assay”: ECO also conveys which assay was used, for example “microscopy” or “RNA interference”. In addition to evidence terms, the ECO ontology provides *assertion terms* in which the nature of the assay is given. For example, an enzyme-linked immunosorbent assay (ELISA) provides quantitative protein data *in vitro* while an immunogold assay may provide the same information, and cellular localization information *in situ*. We manually assigned Evidence Codes Ontology (ECO) assertion and evidence terms to the top-50 articles. The assignment is shown in detail in Table S2 in Text S1. Table S3 in Text S1 shows the sorted count of ECO terms in the top-50 papers.

The most frequent ECO term used is ECO:0000160 “protein separation followed by fragment identification evidence”: this fits the 27 papers that essentially describe mass-spectrometry studies. Consequently this means that the assignment procedure is limited to the cellular compartments that can be identified with the fractionation methods used. So while Cellular Component is the most frequent annotation used, fractionation and mass-spectrometry is the most common method used to localize proteins in subcellular compartments. A notable exception to the use of fractionation and MS for protein localization is in the top annotating article [9], which uses microscopy for subcellular localization.

The second most frequent experimental ECO term is “Imaging assay evidence” (ECO:000044). Several types of studies fall under this ECO. Those include microscopy, RNAi, some of the mass-spectrometry studies that used microscopy, and a yeast-2-hybrid study. As imaging information is used in a variety of studies, this ECO term is not informative of the chief method used in any study, but rather the importance of imaging assays in a variety of methods. The third most frequent experimental ECO term used was “Cell fractionation evidence” which is closely associated with the top term, “Protein separation followed by fragment identification evidence.” The fourth and fifth most frequent ECO term used were “loss-of-function mutant phenotype evidence” (ECO:0000016) and “RNAi evidence” (ECO:000019). These two terms are also closely associated, in RNAi whole-genome gene knockdowns in *C. elegans*, *D. melanogaster* and one in *C. albicans*. RNAi experiments use targeted dsRNA which is delivered to the organism and silences specific genes. Typically the experiments here used libraries of RNAi targeted to the whole exome (for example [10]–[13]). The phenotypes searched for were mostly associated with embryonic and post-embryonic development. Some studies focused on mitotic spindle assembly [14], lipid storage [15] and endocytic traffic [16]. One study used RNAi to identify mitochondrial protein localization [17]. These studies mostly use the same RNAi libraries, and target the whole *C. elegans* genome using common data resources. Hence the large redundancy observed for *C. elegans* in Table 2. It should be noted that all experiments are associated with computational ECO terms, which describe sequence similarity and motif recognition techniques used to identify the sequences found: “sequence similarity evidence”, “transmembrane domain prediction evidence”, “protein BLAST evidence” etc. Computational terms are bolded in Table S2 in Text S1. A strong reliance on computational annotation is therefore an integral part of high throughput experiments. Furthermore, computational annotation here is not used directly for functional annotation, but rather for identifying the protein by a sequence or motif similarity search. The third most frequently used assertion in the top experimental articles was not an experimental assertion, but rather a computational one: the term ECO:00053 “computational combinatorial evidence” is defined as “A type of combinatorial analysis where data are combined and evaluated by an algorithm.” This is not a computational prediction *per se*, but rather a computational combination of several experimental lines of evidence used in a article.

## Discussion

We have identified several annotation biases in GO annotations provided by the GO consortium. These biases stem from the uneven number of annotations produced by different types of experiments. It is clear that results from high-throughput experiments contribute substantially to the function annotation landscape, as up to 20% of experimentally annotated proteins are annotated by high-throughput assays. At the same time, high throughput experiments produce less information per protein than moderate-, low- and single- throughput experiments as evidenced by the type of GO terms produced in the Molecular Function and Biological Process ontologies. Furthermore, the number of total GO terms used in the high-throughput experiments is much lower than that used in low and medium throughput experiments. Therefore, while high throughput experiments provide a high coverage of protein function space, it is the low throughput experiments that provide more specific information, as well as a larger diversity of terms.

We have also identified several types of biases that are contributed by high throughput experiments. First, there is the enrichment of low information-content GO terms, which means that our understanding of the protein function as provided by high-throughput experiments is more limited than that provided by low-throughput experiments. Second, there is the small number of terms used, when considering the large number of proteins that are being annotated. Third is the general ontology bias towards the cellular component ontology and, to a lesser extent, the Biological Process ontology: there are very few articles that deal with the Molecular Function ontology. These biases all stem from the inherent capabilities and limitations of the hight-throughput experiments. A fourth, related bias is the organism studied: taken together, studies of *C. elegans* and *A. thaliana* studies comprise 36 of the top-50 annotating articles, or 72%.

### Information Capture and Scope of GO

We have discussed the information loss that is characteristic of high-throughput experiments, as shown in Figure 4. However, another reason for information loss is the inability to capture certain types of information using the Gene Ontology. GO is purposefully limited to three aspects (MF, BP and CC) of biological function, which are assigned per protein. However, other aspects of function may emerge from experiments. Of note is the study, “Proteome survey reveals modularity of the yeast cell machinery” [9]. In this study, the information produced was primarily of protein complexes, and the relationship to cellular compartmentalization and biological networks. At the same time, the only GO term captured in the curation of proteins from this study was “protein binding”. Some, but not all of this information can be captured more specifically using the children of the term “protein binding”, but such a process is arguably laborious by manual curation of the information from a high throughput article. Furthermore, the main information conveyed by this article, namely the types of protein complexes discovered and how they relate to cellular networks, is outside the scope of GO. It is important to realize that while high-throughput experiments do convey less information per protein within the functional scope as defined by GO, they still convey composite information such as possible pathway mappings - information which needs to be captured into annotation databases by means other than GO. In the example above, the information can be captured by a protein interaction database, but not by GO terms. Methods such as the Statistical Tracking of Ontological Phrases [18] can help in selecting the appropriate ontology for better information capture.

### Conclusions

Taken together, the annotation trends in high-throughput studies affect our understanding of protein function space. This, in turn, affects our ability to properly understand the connection between predictors of protein function and the actual function - the hallmark of computational function annotation. As a dramatic example, during the 2011 Critical Assessment of Function Annotation experiment [19] it was noticed that roughly 20% of the proteins participating in the challenge and annotated with the Molecular Function Ontology were annotated as “protein binding”, a GO term that conveys little information. Furthermore, it was shown that the major contribution of “protein binding” term to the CAFA challenge data set was due to high-throughput assays. This illustrates how the concentration of a large number of annotations in a small number of studies provides only a partial picture of the function of these proteins. As we have seen, the picture provided from high throughput experiments is mainly of: 1) subcellular localization cell fractionation and MS based localization and 2) developmental phenotypes. While these data are important, we should be mindful of this bias when examining protein function in the database, even those annotations deemed to be of high quality, those with experimental verification. Furthermore, such a large bias in prior probabilities can adversely affect programs employing prior probabilities, as most machine-learning programs do. If the training set for these programs has included a disproportional number of annotations by high-throughput experiments, the results these programs provide will be strongly biased towards a few frequent and shallow GO terms.

To remedy the bias created by high throughput annotations, the provenance of annotations should be described in more detail by curators and annotation software. Many function annotation algorithms rely on homology transfer as part of their pipeline to annotate query sequences [1], [19]. Knowing the annotation provenance, including the number of proteins annotated by the original paper can create less biased benchmarks or otherwise incorporate that information into the annotation procedure. The ECO ontology can be used to determine the source of the annotation, and the user or the algorithm can decide whether to rely upon any combinations of “throughputness” and experimental type. Of course, such approaches should be taken cautiously, as sweeping measures can cause the unintended loss of information. We hereby call upon the communities of annotators, computational biologists and experimental biologists to be mindful of the phenomenon of the experimental biases described in this study, and to work to understand its implications and impact.

## Methods

We used the UniProt-GOA database from December 2011. Data analyses were performed using Python scripts. The following tools were used in the analyses: Biopython [20], matplotlib [21]. ECO terms classifying the proteins in the top 50 experiments were assigned to the proteins manually after reading the articles. All data and scripts are available on: http://github.com/FriedbergLab/Uniprot-Bias/ and on http://datadryad.org (the latter upon acceptance).

### Use of GO Evidence Codes

Proteins in UniProt-GOA are annotated with one or more GO terms using a procedure described in Dimmer *et al.* (2012). Briefly, this procedure consists of six steps which include sequence curation, sequence motif analyses, literature-based curation, reciprocal BLAST [22] searches, attribution of all resources leading to the included findings, and quality assurance. If the annotation source is a research article, the attribution includes its PubMed ID. For each GO term associated with a protein, there is also an *evidence code* which the curator assigns to explain how the association between the protein and the GO term was made. Experimental evidence codes include such terms as: *Inferred by Direct Assay* (IDA) which indicates that “a direct assay was carried out to determine the function, process, or component indicated by the GO term” or *Inferred from Physical Interaction* (IPI) which “Covers physical interactions between the gene product of interest and another molecule.” (All GO evidence code definitions were taken from the GO site, geneontology.org.) Computational evidence codes include terms such as *Inferred from Sequence or Structural Similarity* (ISS) and *Inferred from Sequence Orthology* (ISO). Although the evidence in computational evidence codes is non-experimental, the proteins annotated with these evidence codes are still assigned by a curator, rendering a degree of human oversight. Finally, there are also computational, non-experimental evidence codes, the most prevalent being *Inferred from Electronic Annotation* (IEA) which is “used for annotations that depend directly on computation or automated transfer of annotations from a database”. IEA evidence means that the annotation is electronic, and was not made or checked by a person. Different degrees of reliability are associated with different evidence codes, with experimental codes generally considered to be of higher reliability than non-experimental codes. (For details see: http://www.ebi.ac.uk/GOA/ElectronicAnnotationMethods).

### Quantifying GO-term Information

We used two methods to quantify the information given by GO terms. First we used edge counting, where the information contained in a term is dependent on the edge distance of that term from the root. The term “catalytic activity”(one edge distance from the ontology root node) would be less informative than “hydrolase activity” (two edges) and the latter will be less informative than “haloalkane dehalogenase activity” (five edges). We therefore counted edges from the ontology root term to the GO term to determine term information. The larger the number of edges, the more specific -and therefore informative- is the annotation. In cases where several paths lead from the root to the examined GO term, we used the minimal path. We did so for all the annotating articles split into groups by the number of proteins each article annotates.

While edge counting provides a measure of term-specificity, this measure is imperfect. The reason is that each of the three GO ontologies is constructed as a directed acyclic graph (DAG) where different areas of the GO DAG have different connectivities, and terms may have different depths unrelated to the intuitive specificity of a term. For example “D-glucose transmembrane transporter activity”, (GO:0055056) is 10 terms deep, while “L-tryptophan transmembrane transporter activity”, (GO:0015196) is fourteen terms deep. It is hard to discern whether these differences are meaningful. For this reason, information content, the logarithm of the inverse of the GO term frequency in the corpus, is generally accepted as a measure of GO term information content [23], [24]. To account for the possible bias created by the GO-DAG structure, we also used the log-frequency of the terms in the experimentally annotated proteins in Uniprot-GOA. However, the log-frequency measure is also imperfect because, as we see throughout this study, a GO term's frequency may be heavily influenced by the top annotating articles, injecting a circularity problem into the use of this metric. Since no single metric for measuring the information conveyed by a GO term is wholly satisfactory, we report the results from both edge-counting and information-content.

### Annotation Consistency

To examine annotation consistency, we employed the following method: given a protein 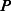, let 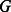 be the terminal (leaf) GO terms 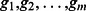 that annotate that protein in all top-50 articles for a single ontology 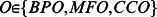. The count of each of these GO terms per protein per ontology is 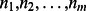 with 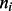 being the number of times GO term 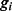 annotates protein 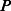.

The number of total annotations for a protein in an ontology is 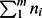. The *maximum annotation consistency* for protein 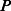 in ontology 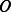 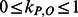 is calculated as:
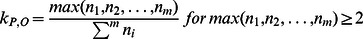



For example, the protein “Oleate activated transcription factor 3” (UniProtID: P36023) in *S. cerevisiae* is annotated four times by three articles using the Cellular Component ontology, as shown in Table 5. The annotation consistency for P36023 is therefore the maximum count of identical GO terms (*mitochondrion*, 2), divided by the total number of annotations, 4: 0.5.

**Table 5:** Annotation Consistency Example.

PubMedID	UniProt ID	Ontology	GO term	description
14562095	P36023	CCO	GO:0005634	nucleus
14562095	P36023	CCO	GO:0005737	cytoplasm
16823961	P36023	CCO	GO:0005739	mitochondrion
14576278	P36023	CCO	GO:0005739	mitochondrion

Example of annotation consistency of a single protein in four publications. See Methods for details.

When choosing a measure for annotation consistency, we favored a simple and interpretable measure. We therefore examined identity among leaf terms only, rather than use a more complex comparison of multiple subgraphs in the GO ontology DAG (Directed Acyclic Graph). Doing so without manual curation is unreliable, and may skew the perception of similarity [25].

## Supporting Information

Text S1 This file contains supplementary tables. **Table S1:** The top 50 annotating articles. N: article rank; Proteins: number of proteins annotated in this article; Annotations: number of annotating GO terms; Species: annotated species; ref. annotating article; MFO/BPO/CCO: number of proteins annotated in the Molecular Function, Biological Process and Cellular Component ontologies, respectively. **Table S2:** The Top-50 studies and the ECO terms we have assigned to them. PMID: Articles' PubMed ID; ECO terms/ECO ID's: terms and ID's we assigned to the articles. **Table S3:** ECO terms were assigned by us to the top-50 annotating papers. The table entries are ranked by the frequency of the assignments, i.e. 27 papers are assigned with term ECO:0000160, 21 were assigned ECO:0000004, etc. Entries in boldface are for computational methods, which were used in many papers in combination with experimental methods to assign function. Table S2 lists the ECO terms.
(PDF)
