# Supplementary material for: Biases in the Experimental Annotations of Protein Function and Their Effect on Our Understanding of Protein Function Space
Source: PLoS Comput Biol. 2013 May 30;9(5):e1003063. doi: 10.1371/journal.pcbi.1003063 (PMC3667760; doi:10.1371/journal.pcbi.1003063)
Supplement: Text S1 — This file contains supplementary tables. Table S1: The top 50 annotating articles. N: article rank; Proteins: number of proteins annotated in this article; Annotations: number of annotating GO terms; Species: annotated species; ref. annotating article; MFO/BPO/CCO: number of proteins annotated in the Molecular Function, Biological Process and Cellular Component ontologies, respectively. Table S2: The Top-50 studies and the ECO terms we have assigned to them. PMID: Articles' PubMed ID; ECO terms/ECO ID's: terms and ID's we assigned to the articles. Table S3: ECO terms were assigned by us to the top-50 annotating papers. The table entries are ranked by the frequency of the assignments, i.e. 27 papers are assigned with term ECO:0000160, 21 were assigned ECO:0000004, etc. Entries in boldface are for computational methods, which were used in many papers in combination with experimental methods to assign function. Table S2 lists the ECO terms. (PDF) [file pcbi.1003063.s001.pdf]

Text S1

Supplementary Tables Legends

Table S1: The top 50 annotating articles.

N: article rank; **Proteins**: number of proteins annotated in this article; **Annotations**: number of annotating GO terms; **Species**: annotated species; **ref.** annotating article; **MFO/BPO/CCO**: number of proteins annotated in the Molecular Function, Biological Process and Cellular Component ontologies, respectively.

Table S2: The Top-50 studies and the ECO terms we have assigned to them.

PMID: Articles' PubMed ID; **ECO terms/ECO ID's**: terms and ID's we assigned to the articles.

Table S3: ECO terms were assigned by us to the top-50 annotating papers.

The table entries are ranked by the frequency of the assignments, i.e. 27 papers are assigned with term ECO:0000160, 21 were assigned ECO:0000004, etc. Entries in **boldface** are for computational methods, which were used in many papers in combination with experimental methods to assign function. Table S2 lists the ECO terms.

Supplementary Tables

Table S1. Top 50 Annotating Articles

N	Proteins	Annotations	Species	ref.	MFO	BPO	CCO
1	4937	11050	*H. sapiens*	[1]	0	0	11050
2	4247	7046	*S. pombe*	[2]	0	0	7046
3	2412	2412	*H. sapiens*	[3]	0	0	2412
4	1791	5918	*C. elegans*	[4]	0	5918	0
5	1406	1863	*S. cerevisiae*	[5]	0	0	1863
6	1251	1251	*A. thaliana*	[6]	0	0	1251
7	1205	1476	*C. elegans*	[7]	0	1476	0
8	1186	1213	*M. musculus*	[8]	0	0	1213
9	1136	1136	*A. thaliana*	[9]	0	0	1136
10	1101	2269	*C. elegans*	[10]	0	2269	0
11	1043	1365	*M. tuberculosis*	[11]	0	0	1365
12	1041	1041	*A. thaliana*	[12]	0	0	1041
13	865	1533	*C. elegans*	[13]	0	1533	0
14	845	845	*S. cerevisiae*	[14]	0	0	845
15	784	784	*A. thaliana*	[15]	0	0	784
16	735	735	*M. tuberculosis*	[16]	0	0	735
17	724	882	*A. thaliana*	[17]	0	0	882
18	634	634	*A. thaliana*	[18]	0	0	634
19	613	613	Mycobacter sp.	[19]	0	613	0
20	607	661	*C. elegans*	[20]	0	659	2

Continued on next page

N	Proteins	Annotations	Species	ref.	MFO	BPO	CCO
21	577	577	*A. thaliana*	[21]	0	0	577
22	553	884	*C. elegans*	[22]	0	884	0
23	516	5972	*C. elegans*	[23]	0	5972	0
24	503	503	*S. cerevisiae*	[24]	0	0	503
25	498	638	*S. cerevisiae*	[25]	638	0	0
26	479	848	*C. elegans*	[26]	0	848	0
27	465	468	*H. sapiens*	[27]	0	0	468
28	436	436	*A. thaliana*	[28]	0	0	436
29	430	513	*A. thaliana*	[29]	0	0	513
30	413	456	*D. melanogaster*	[30]	0	39	417
31	401	401	*A. thaliana*	[31]	0	0	401
32	392	392	*A. thaliana*	[32]	0	0	392
33	392	639	*C. elegans*	[33]	0	639	0
34	383	917	*C. elegans*	[34]	0	917	0
35	380	380	*A. thaliana*	[35]	0	0	380
36	375	375	*M. musculus*	[36]	0	0	375
37	343	509	*H. sapiens*	[37]	509	0	0
38	338	338	Ddiscoideum	[38]	0	0	338
39	328	328	*A. thaliana*	[39]	0	0	328
40	319	329	*C. albicans*	[40]	1	328	0
41	305	312	*A. thaliana*	[41]	0	0	312
42	290	331	*S. cerevisiae*	[42]	0	0	331

Continued on next page

**N**	**Proteins**	**Annotations**	**Species**	**ref.**	**MFO**	**BPO**	**CCO**
43	285	761	*C. elegans*	[43]	0	761	0
44	283	499	*C. elegans*	[44]	0	499	0
45	266	433	*M. musculus*	[45]	433	0	0
46	260	260	*A. thaliana*	[46]	0	260	0
47	258	259	*S. pombe*	[47]	0	259	0
48	244	397	*D. melanogaster*	[48]	0	367	30
49	242	397	*D. melanogaster*	[49]	0	0	397
50	241	263	*A. thaliana*	[50]	0	0	263

Table S2. ECO Terms Assigned to Top-50 Papers

PMID	Ref	ECO terms/ECO ID's
18029348	[1]	imaging assay evidence/ECO:0000324 immunofluorescence evidence/ECO:0000007 immunolocalization evidence/ECO:0000087
16823372	[2]	imaging assay evidence/ECO:0000324 yellow fluorescent protein fusion protein localization evidence/ECO:0000128 enzyme inhibition experiment evidence/ECO:0000184
18614015	[3]	imaging assay evidence/ECO:0000324 protein separation followed by fragment identification evidence/ECO:0000160 sequence similarity evidence/ECO:0000044 cell fractionation evidence/ECO:0000004 GFP fusion protein localization evidence/ECO:0000126 computational combinatorial evidence/ECO:0000053 motif similarity evidence/ECO:0000028 targeting sequence prediction evidence/ECO:0000081 protein BLAST evidence/ECO:0000208
14551910	[4]	imaging assay evidence/ECO:0000324 RNAi evidence/ECO:0000019 loss-of-function mutant phenotype evidence/ECO:0000016 nucleotide BLAST evidence/ECO:0000207 sequence alignment evidence/ECO:0000200
14562095	[5]	imaging assay evidence/ECO:0000324 GFP fusion protein localization evidence/ECO:0000126 fusion protein localization evidence/ECO:0000124 affinity chromatography evidence/ECO:0000079

Continued on next page

PMID	Ref	ECO terms/ECO ID's
18431481	[6]	protein separation followed by fragment identification evidence/ECO:0000160 targeting sequence prediction evidence/ECO:0000081 cell fractionation evidence/ECO:0000004 sequence similarity evidence/ECO:0000044 imported information/ECO:0000311
15791247	[7]	imaging assay evidence/ECO:0000324 RNAi evidence/ECO:0000019 loss-of-function mutant phenotype evidence/ECO:0000016 protein BLAST evidence/ECO:0000208
14651853	[8]	protein separation followed by fragment identification evidence/ECO:0000160 cell fractionation evidence/ECO:0000004 targeting sequence prediction evidence/ECO:0000081 sequence similarity evidence/ECO:0000044 protein BLAST evidence/ECO:0000208 nucleotide BLAST evidence/ECO:0000207 Affymetrix array experiment evidence/ECO:0000101 imported information/ECO:0000311
17317660	[9]	protein separation followed by fragment identification evidence/ECO:0000160 cell fractionation evidence/ECO:0000004 transmembrane domain prediction evidence/ECO:0000083 sequence similarity evidence/ECO:0000044

Continued on next page

PMID	Ref	ECO terms/ECO ID's
12529635	[10]	imaging assay evidence/ECO:0000324 RNAi evidence/ECO:0000019 loss-of-function mutant phenotype evidence/ECO:0000016 motif similarity evidence/ECO:0000028 protein BLAST evidence/ECO:0000208 nucleotide BLAST evidence/ECO:0000207 computational combinatorial evidence/ECO:0000053
15525680	[11]	protein separation followed by fragment identification evidence/ECO:0000160 cell fractionation evidence/ECO:0000004 transmembrane domain prediction evidence/ECO:0000083 sequence similarity evidence/ECO:0000044 computational combinatorial evidence/ECO:0000053 biological system reconstruction/ECO:0000088 imported information/ECO:0000311 protein BLAST evidence/ECO:0000208
21166475	[12]	protein separation followed by fragment identification evidence/ECO:0000160 cell fractionation evidence/ECO:0000004 sequence similarity evidence/ECO:0000044 computational combinatorial evidence/ECO:0000053 imported information/ECO:0000311 transmembrane domain prediction evidence/ECO:0000083 sequence alignment evidence/ECO:0000200 motif similarity evidence/ECO:0000028
15489339	[13]	imaging assay evidence/ECO:0000324 RNAi evidence/ECO:0000019 loss-of-function mutant phenotype evidence/ECO:0000016 nucleotide BLAST evidence/ECO:0000207

Continued on next page

PMID	Ref	ECO terms/ECO ID's
16823961	[14]	protein separation followed by fragment identification evidence/ECO:0000160 cell fractionation evidence/ECO:0000004 sequence similarity evidence/ECO:0000044 imported information/ECO:0000311
21533090	[15]	protein separation followed by fragment identification evidence/ECO:0000160 cell fractionation evidence/ECO:0000004 sequence similarity evidence/ECO:0000044 imported information/ECO:0000311 computational combinatorial evidence/ECO:0000053 transmembrane domain prediction evidence/ECO:0000083 sequence alignment evidence/ECO:0000200 motif similarity evidence/ECO:0000028 targeting sequence prediction evidence/ECO:0000081
14532352	[16]	protein separation followed by fragment identification evidence/ECO:0000160 cell fractionation evidence/ECO:0000004 sequence similarity evidence/ECO:0000044 transmembrane domain prediction evidence/ECO:0000083
20061580	[17]	protein separation followed by fragment identification evidence/ECO:0000160 cell fractionation evidence/ECO:0000004 sequence similarity evidence/ECO:0000044 transmembrane domain prediction evidence/ECO:0000083 imported information/ECO:0000311 targeting sequence prediction evidence/ECO:0000081 protein expression level evidence/ECO:0000046

Continued on next page

PMID	Ref	ECO terms/ECO ID's
15028209	[18]	protein separation followed by fragment identification evidence/ECO:0000160 cell fractionation evidence/ECO:0000004 sequence similarity evidence/ECO:0000044 targeting sequence prediction evidence/ECO:0000081 Affymetrix array experiment evidence/ECO:0000101 protein expression level evidence/ECO:0000046 protein BLAST evidence/ECO:0000208 computational combinatorial evidence/ECO:0000053 motif similarity evidence/ECO:0000028 transmembrane domain prediction evidence/ECO:0000083
12657046	[19]	mutant phenotype evidence/ECO:0000015 nucleic acid hybridization evidence/ECO:0000026 imported information/ECO:0000311 sequence similarity evidence/ECO:0000044 combinatorial evidence/ECO:0000212
17704769	[20]	imaging assay evidence/ECO:0000324 RNAi evidence/ECO:0000019 loss-of-function mutant phenotype evidence/ECO:0000016
17432890	[21]	protein separation followed by fragment identification evidence/ECO:0000160 cell fractionation evidence/ECO:0000004 sequence similarity evidence/ECO:0000044 transmembrane domain prediction evidence/ECO:0000083 imported information/ECO:0000311 targeting sequence prediction evidence/ECO:0000081 protein BLAST evidence/ECO:0000208 computational combinatorial evidence/ECO:0000053

Continued on next page

PMID	Ref	ECO terms/ECO ID's
11231151	[22]	imaging assay evidence/ECO:0000324 RNAi evidence/ECO:0000019 loss-of-function mutant phenotype evidence/ECO:0000016
17417969	[23]	imaging assay evidence/ECO:0000324 RNAi evidence/ECO:0000019 loss-of-function mutant phenotype evidence/ECO:0000016
14576278	[24]	protein separation followed by fragment identification evidence/ECO:0000160 cell fractionation evidence/ECO:0000004 sequence similarity evidence/ECO:0000044 transmembrane domain prediction evidence/ECO:0000083
16429126	[25]	protein separation followed by fragment identification evidence/ECO:0000160 sequence similarity evidence/ECO:0000044 affinity chromatography evidence/ECO:0000079 protein BLAST evidence/ECO:0000208 imported information/ECO:0000311
21529718	[26]	imaging assay evidence/ECO:0000324 RNAi evidence/ECO:0000019 loss-of-function mutant phenotype evidence/ECO:0000016 computational combinatorial evidence/ECO:0000053
11256614	[27]	GFP fusion protein localization evidence/ECO:0000126 yellow fluorescent protein fusion protein localization evidence/ECO:0000128 imaging assay evidence/ECO:0000324 motif similarity evidence/ECO:0000028 protein BLAST evidence/ECO:0000208 nucleotide BLAST evidence/ECO:0000207

Continued on next page

PMID	Ref	ECO terms/ECO ID's
17644812	[28]	protein separation followed by fragment identification evidence/ECO:0000160 cell fractionation evidence/ECO:0000004 sequence similarity evidence/ECO:0000044 transmembrane domain prediction evidence/ECO:0000083 targeting sequence prediction evidence/ECO:0000081 computational combinatorial evidence/ECO:0000053
16618929	[29]	protein separation followed by fragment identification evidence/ECO:0000160 cell fractionation evidence/ECO:0000004 sequence similarity evidence/ECO:0000044 transmembrane domain prediction evidence/ECO:0000083
18433294	[30]	protein separation followed by fragment identification evidence/ECO:0000160 cell fractionation evidence/ECO:0000004 sequence similarity evidence/ECO:0000044 imaging assay evidence/ECO:0000324 RNAi evidence/ECO:0000019 loss-of-function mutant phenotype evidence/ECO:0000016 immunofluorescence evidence/ECO:0000007
17151019	[31]	protein separation followed by fragment identification evidence/ECO:0000160 cell fractionation evidence/ECO:0000004 sequence similarity evidence/ECO:0000044 imported information/ECO:0000311 transmembrane domain prediction evidence/ECO:0000083

Continued on next page

PMID	Ref	ECO terms/ECO ID's
14671022	[32]	protein separation followed by fragment identification evidence/ECO:0000160 cell fractionation evidence/ECO:0000004 sequence similarity evidence/ECO:0000044 protein BLAST evidence/ECO:0000208 targeting sequence prediction evidence/ECO:0000081
12529643	[33]	imaging assay evidence/ECO:0000324 RNAi evidence/ECO:0000019 loss-of-function mutant phenotype evidence/ECO:0000016
12445391	[34]	imaging assay evidence/ECO:0000324 RNAi evidence/ECO:0000019 loss-of-function mutant phenotype evidence/ECO:0000016 BLAST evidence/ECO:0000206
15539469	[35]	protein separation followed by fragment identification evidence/ECO:0000160 cell fractionation evidence/ECO:0000004 sequence similarity evidence/ECO:0000044 targeting sequence prediction evidence/ECO:0000081 transmembrane domain prediction evidence/ECO:0000083 motif similarity evidence/ECO:0000028 protein BLAST evidence/ECO:0000208 computational combinatorial evidence/ECO:0000053
12865426	[36]	protein separation followed by fragment identification evidence/ECO:0000160 cell fractionation evidence/ECO:0000004 sequence similarity evidence/ECO:0000044 transmembrane domain prediction evidence/ECO:0000083

Continued on next page

PMID	Ref	ECO terms/ECO ID's
16189514	[37]	yeast 2-hybrid evidence/ECO:0000068 imaging assay evidence/ECO:0000324 motif similarity evidence/ECO:0000028 co-purification evidence/ECO:0000022 combinatorial evidence/ECO:0000212
20422638	[38]	protein separation followed by fragment identification evidence/ECO:0000160 cell fractionation evidence/ECO:0000004 sequence similarity evidence/ECO:0000044 combinatorial evidence/ECO:0000212
12938931	[39]	protein separation followed by fragment identification evidence/ECO:0000160 cell fractionation evidence/ECO:0000004 sequence similarity evidence/ECO:0000044 nucleotide BLAST evidence/ECO:0000207 imported information/ECO:0000311 transmembrane domain prediction evidence/ECO:0000083
16336044	[40]	imaging assay evidence/ECO:0000324 RNAi evidence/ECO:0000019 loss-of-function mutant phenotype evidence/ECO:0000016
18633119	[41]	protein separation followed by fragment identification evidence/ECO:0000160 cell fractionation evidence/ECO:0000004 Western blot evidence/ECO:0000112
11914276	[42]	imaging assay evidence/ECO:0000324 immunofluorescence evidence/ECO:0000007 epitope-tagged protein immunolocalization evidence/ECO:0000092 transmembrane domain prediction evidence/ECO:0000083 imported information/ECO:0000311

Continued on next page

PMID	Ref	ECO terms/ECO ID's
11099033	[43]	imaging assay evidence/ECO:0000324 RNAi evidence/ECO:0000019 loss-of-function mutant phenotype evidence/ECO:0000016 protein BLAST evidence/ECO:0000208 computational combinatorial evidence/ECO:0000053
11099034	[44]	imaging assay evidence/ECO:0000324 RNAi evidence/ECO:0000019 loss-of-function mutant phenotype evidence/ECO:0000016 nucleotide BLAST evidence/ECO:0000207 protein BLAST evidence/ECO:0000208
11591653	[45]	hybrid interaction evidence/ECO:0000025 imaging assay evidence/ECO:0000324
16502469	[46]	protein separation followed by fragment identification evidence/ECO:0000160 sequence similarity evidence/ECO:0000044 protein BLAST evidence/ECO:0000208 Northern assay evidence/ECO:0000106 reverse transcription polymerase chain reaction transcription evidence/ECO:0000108
12529438	[47]	microarray RNA expression level evidence/ECO:0000104 sequence orthology evidence used in manual assertion/ECO:0000266 motif similarity evidence/ECO:0000028
17412918	[48]	RNAi evidence/ECO:0000019 loss-of-function mutant phenotype evidence/ECO:0000016 imaging assay evidence/ECO:0000324

Continued on next page

PMID	Ref	ECO terms/ECO ID's
18981222	[49]	protein separation followed by fragment identification evidence/ECO:0000160 sequence similarity evidence/ECO:0000044 protein BLAST evidence/ECO:0000208 in vitro assay evidence/ECO:0000181 affinity chromatography evidence/ECO:0000079 imaging assay evidence/ECO:0000324 mutant phenotype evidence/ECO:0000015
1p6287169	[50]	protein separation followed by fragment identification evidence/ECO:0000160 sequence similarity evidence/ECO:0000044 transmembrane domain prediction evidence/ECO:0000083 sequence alignment evidence/ECO:0000200 computational combinatorial evidence/ECO:0000053 motif similarity evidence/ECO:0000028 targeting sequence prediction evidence/ECO:0000081

Table S3. Count of ECO terms in top-50 papers

N	ECO term	ECO ID	Articles
1	protein separation followed by fragment identification evidence	ECO:0000160	27
2	**sequence similarity evidence**	ECO:0000044	27
3	imaging assay evidence	ECO:0000324	24
4	cell fractionation evidence	ECO:0000004	23
5	**transmembrane domain prediction evidence**	ECO:0000083	17
6	loss-of-function mutant phenotype evidence	ECO:0000016	15
7	**protein BLAST evidence**	ECO:0000208	15
8	RNAi evidence	ECO:0000019	15
9	imported information	ECO:0000311	13
10	**computational combinatorial evidence**	ECO:0000053	11
11	**targeting sequence prediction evidence**	ECO:0000081	11
12	**motif similarity evidence**	ECO:0000028	10
13	**nucleotide BLAST evidence**	ECO:0000207	7
14	**sequence alignment evidence**	ECO:0000200	4
15	GFP fusion protein localization evidence	ECO:0000126	3
16	immunofluorescence evidence	ECO:0000007	3
17	affinity chromatography evidence	ECO:0000079	3
18	**computational combinatorial evidence**	ECO:0000053	2
19	Affymetrix array experiment evidence	ECO:0000101	2

Continued on next page

N	ECO term	ECO ID	Articles
20	protein expression level evidence	ECO:0000046	2
21	mutant phenotype evidence	ECO:0000015	2
22	combinatorial evidence	ECO:0000212	2
23	co-purification evidence	ECO:0000022	1
24	epitope-tagged protein immunolocalization evidence	ECO:0000092	1
25	**sequence orthology evidence used in manual assertion**	ECO:0000266	1
26	YFP fusion protein localization evidence	ECO:0000128	2
27	in vitro assay evidence	ECO:0000181	1
28	biological system reconstruction	ECO:0000088	1
29	reverse transcription polymerase chain reaction transcription evidence	ECO:0000108	1
30	Northern assay evidence	ECO:0000106	1
31	Western blot evidence	ECO:0000112	1
32	microarray RNA expression level evidence	ECO:0000104	1
33	fusion protein localization evidence	ECO:0000124	1
34	**BLAST evidence**	ECO:0000206	1
35	nucleic acid hybridization evidence	ECO:0000026	1
36	enzyme inhibition experiment evidence	ECO:0000184	1
37	immunolocalization evidence	ECO:0000087	1
38	hybrid interaction evidence	ECO:0000025	1
39	yeast 2-hybrid evidence	ECO:0000068	1

References

1. Barbe L, Lundberg E, Oksvold P, Stenius A, Lewin E, et al. (2008) Toward a confocal subcellular atlas of the human proteome. Mol Cell Proteomics 7: 499–508.

2. Matsuyama A, Arai R, Yashiroda Y, Shirai A, Kamata A, et al. (2006) ORFeome cloning and global analysis of protein localization in the fission yeast Schizosaccharomyces pombe. Nat Biotechnol 24: 841–847.

3. Pagliarini DJ, Calvo SE, Chang B, Sheth SA, Vafai SB, et al. (2008) A mitochondrial protein compendium elucidates complex I disease biology. Cell 134: 112–123.

4. Simmer F, Moorman C, van der Linden AM, Kuijk E, van den Berghe PV, et al. (2003) Genome-wide RNAi of C. elegans using the hypersensitive rrf-3 strain reveals novel gene functions. PLoS Biol 1: E12.

5. Huh WK, Falvo JV, Gerke LC, Carroll AS, Howson RW, et al. (2003) Global analysis of protein localization in budding yeast. Nature 425: 686–691.

6. Zybailov B, Rutschow H, Friso G, Rudella A, Emanuelsson O, et al. (2008) Sorting signals, N-terminal modifications and abundance of the chloroplast proteome. PLoS ONE 3: e1994.

7. Sonnichsen B, Koski LB, Walsh A, Marschall P, Neumann B, et al. (2005) Full-genome RNAi profiling of early embryogenesis in Caenorhabditis elegans. Nature 434: 462–469.

8. Mootha VK, Bunkenborg J, Olsen JV, Hjerrild M, Wisniewski JR, et al. (2003) Integrated analysis of protein composition, tissue diversity, and gene regulation in mouse mitochondria. Cell 115: 629–640.

9. Benschop JJ, Mohammed S, O'Flaherty M, Heck AJ, Slijper M, et al. (2007) Quantitative phosphoproteomics of early elicitor signaling in Arabidopsis. Mol Cell Proteomics 6: 1198–1214.

10. Kamath RS, Fraser AG, Dong Y, Poulin G, Durbin R, et al. (2003) Systematic functional analysis of the Caenorhabditis elegans genome using RNAi. Nature 421: 231–237.

11. Mawuenyega KG, Forst CV, Dobos KM, Belisle JT, Chen J, et al. (2005) Mycobacterium tuberculosis functional network analysis by global subcellular protein profiling. Mol Biol Cell 16: 396–404.

12. Ito J, Batth TS, Petzold CJ, Redding-Johanson AM, Mukhopadhyay A, et al. (2011) Analysis of the Arabidopsis cytosolic proteome highlights subcellular partitioning of central plant metabolism. J Proteome Res 10: 1571–1582.

13. Rual JF, Ceron J, Koreth J, Hao T, Nicot AS, et al. (2004) Toward improving Caenorhabditis elegans phenome mapping with an ORFeome-based RNAi library. Genome Res 14: 2162–2168.

14. Reinders J, Zahedi RP, Pfanner N, Meisinger C, Sickmann A (2006) Toward the complete yeast mitochondrial proteome: multidimensional separation techniques for mitochondrial proteomics. J Proteome Res 5: 1543–1554.

15. Fernandez-Calvino L, Faulkner C, Walshaw J, Saalbach G, Bayer E, et al. (2011) Arabidopsis plasmodesmal proteome. PLoS ONE 6: e18880.

16. Gu S, Chen J, Dobos KM, Bradbury EM, Belisle JT, et al. (2003) Comprehensive proteomic profiling of the membrane constituents of a Mycobacterium tuberculosis strain. Mol Cell Proteomics 2: 1284–1296.

17. Ferro M, Brugiere S, Salvi D, Seigneurin-Berny D, Court M, et al. (2010) ATCHLORO, a comprehensive chloroplast proteome database with subplastidial localization and curated information on envelope proteins. Mol Cell Proteomics 9: 1063–1084.

18. Kleffmann T, Russenberger D, von Zychlinski A, Christopher W, Sjolander K, et al. (2004) The Arabidopsis thaliana chloroplast proteome reveals pathway abundance and novel protein functions. Curr Biol 14: 354–362.

19. Sassetti CM, Boyd DH, Rubin EJ (2003) Genes required for mycobacterial growth defined by high density mutagenesis. Mol Microbiol 48: 77–84.

20. Balklava Z, Pant S, Fares H, Grant BD (2007) Genome-wide analysis identifies a general requirement for polarity proteins in endocytic traffic. Nat Cell Biol 9: 1066–1073.

21. Mitra SK, Gantt JA, Ruby JF, Clouse SD, Goshe MB (2007) Membrane proteomic analysis of Arabidopsis thaliana using alternative solubilization techniques. J Proteome Res 6: 1933–1950.

22. Maeda I, Kohara Y, Yamamoto M, Sugimoto A (2001) Large-scale analysis of gene function in Caenorhabditis elegans by high-throughput RNAi. Curr Biol 11: 171–176.

23. Ceron J, Rual JF, Chandra A, Dupuy D, Vidal M, et al. (2007) Large-scale RNAi screens identify novel genes that interact with the C. elegans retinoblastoma path-

way as well as splicing-related components with synMuv B activity. BMC Dev Biol 7: 30.

24. Sickmann A, Reinders J, Wagner Y, Joppich C, Zahedi R, et al. (2003) The proteome of Saccharomyces cerevisiae mitochondria. Proc Natl Acad Sci USA 100: 13207–13212.

25. Gavin AC, Aloy P, Grandi P, Krause R, Boesche M, et al. (2006) Proteome survey reveals modularity of the yeast cell machinery. Nature 440: 631–636.

26. Green RA, Kao HL, Audhya A, Arur S, Mayers JR, et al. (2011) A high-resolution C. elegans essential gene network based on phenotypic profiling of a complex tissue. Cell 145: 470–482.

27. Simpson JC, Wellenreuther R, Poustka A, Pepperkok R, Wiemann S (2000) Systematic subcellular localization of novel proteins identified by large-scale cDNA sequencing. EMBO Rep 1: 287–292.

28. Marmagne A, Ferro M, Meinnel T, Bruley C, Kuhn L, et al. (2007) A high content in lipid-modified peripheral proteins and integral receptor kinases features in the arabidopsis plasma membrane proteome. Mol Cell Proteomics 6: 1980–1996.

29. Dunkley TP, Hester S, Shadforth IP, Runions J, Weimar T, et al. (2006) Mapping the Arabidopsis organelle proteome. Proc Natl Acad Sci USA 103: 6518–6523.

30. Hughes JR, Meireles AM, Fisher KH, Garcia A, Antrobus PR, et al. (2008) A microtubule interactome: complexes with roles in cell cycle and mitosis. PLoS Biol 6: e98.

31. Jaquinod M, Villiers F, Kieffer-Jaquinod S, Hugouvieux V, Bruley C, et al. (2007) A proteomics dissection of Arabidopsis thaliana vacuoles isolated from cell culture. Mol Cell Proteomics 6: 394–412.

32. Heazlewood JL, Tonti-Filippini JS, Gout AM, Day DA, Whelan J, et al. (2004) Experimental analysis of the Arabidopsis mitochondrial proteome highlights signaling and regulatory components, provides assessment of targeting prediction programs, and indicates plant-specific mitochondrial proteins. Plant Cell 16: 241–256.

33. Ashrafi K, Chang FY, Watts JL, Fraser AG, Kamath RS, et al. (2003) Genome-wide RNAi analysis of Caenorhabditis elegans fat regulatory genes. Nature 421: 268–272.

34. Piano F, Schetter AJ, Morton DG, Gunsalus KC, Reinke V, et al. (2002) Gene clustering based on RNAi phenotypes of ovary-enriched genes in C. elegans. Curr Biol 12: 1959–1964.

35. Carter C, Pan S, Zouhar J, Avila EL, Girke T, et al. (2004) The vegetative vacuole proteome of Arabidopsis thaliana reveals predicted and unexpected proteins. Plant Cell 16: 3285–3303.

36. Da Cruz S, Xenarios I, Langridge J, Vilbois F, Parone PA, et al. (2003) Proteomic analysis of the mouse liver mitochondrial inner membrane. J Biol Chem 278: 41566–41571.

37. Rual JF, Venkatesan K, Hao T, Hirozane-Kishikawa T, Dricot A, et al. (2005) Towards a proteome-scale map of the human protein-protein interaction network. Nature 437: 1173–1178.

38. Bakthavatsalam D, Gomer RH (2010) The secreted proteome profile of developing Dictyostelium discoideum cells. Proteomics 10: 2556–2559.

39. Froehlich JE, Wilkerson CG, Ray WK, McAndrew RS, Osteryoung KW, et al. (2003) Proteomic study of the Arabidopsis thaliana chloroplastic envelope membrane utilizing alternatives to traditional two-dimensional electrophoresis. J Proteome Res 2: 413–425.

40. Stroschein-Stevenson SL, Foley E, O'Farrell PH, Johnson AD (2006) Identification of Drosophila gene products required for phagocytosis of Candida albicans. PLoS Biol 4: e4.

41. Rutschow H, Ytterberg AJ, Friso G, Nilsson R, van Wijk KJ (2008) Quantitative proteomics of a chloroplast SRP54 sorting mutant and its genetic interactions with CLPC1 in Arabidopsis. Plant Physiol 148: 156–175.

42. Kumar A, Agarwal S, Heyman JA, Matson S, Heidtman M, et al. (2002) Subcellular localization of the yeast proteome. Genes Dev 16: 707–719.

43. Fraser AG, Kamath RS, Zipperlen P, Martinez-Campos M, Sohrmann M, et al. (2000) Functional genomic analysis of C. elegans chromosome I by systematic RNA interference. Nature 408: 325–330.

44. Gonczy P, Echeverri C, Oegema K, Coulson A, Jones SJ, et al. (2000) Functional genomic analysis of cell division in C. elegans using RNAi of genes on chromosome III. Nature 408: 331–336.

45. Suzuki H, Fukunishi Y, Kagawa I, Saito R, Oda H, et al. (2001) Protein-protein interaction panel using mouse full-length cDNAs. Genome Res 11: 1758–1765.

46. Sarry JE, Kuhn L, Ducruix C, Lafaye A, Junot C, et al. (2006) The early responses of Arabidopsis thaliana cells to cadmium exposure explored by protein and metabolite profiling analyses. Proteomics 6: 2180–2198.

47. Chen D, Toone WM, Mata J, Lyne R, Burns G, et al. (2003) Global transcriptional responses of fission yeast to environmental stress. Mol Biol Cell 14: 214–229.

48. Goshima G, Wollman R, Goodwin SS, Zhang N, Scholey JM, et al. (2007) Genes required for mitotic spindle assembly in Drosophila S2 cells. Science 316: 417–421.

49. Herold N, Will CL, Wolf E, Kastner B, Urlaub H, et al. (2009) Conservation of the protein composition and electron microscopy structure of Drosophila melanogaster and human spliceosomal complexes. Mol Cell Biol 29: 281–301.

50. Bayer EM, Bottrill AR, Walshaw J, Vigouroux M, Naldrett MJ, et al. (2006) Arabidopsis cell wall proteome defined using multidimensional protein identification technology. Proteomics 6: 301–311.
